# Neurosensory Evaluation Following Genioplasty: A Case Report

**DOI:** 10.7759/cureus.60709

**Published:** 2024-05-20

**Authors:** Jorge H Fernandes da Rocha, Fernanda C Poscai Ribeiro

**Affiliations:** 1 Facial Surgery, Instituto de Cirurgia Estética Facial (INCEF), São Paulo, BRA; 2 Surgery, Universidade do Oeste Paulista, Guarujá, BRA; 3 Internal Medicine, Universidade do Oeste Paulista, Guarujá, BRA

**Keywords:** mental nerve (mn), fibers assessment, paresthesia, neurosensory testing, genioplasty

## Abstract

Genioplasty is a common procedure in plastic surgery, with various alloplastic biomaterials utilized for chin augmentation. Despite their advantages, complications such as neuropraxia of the mental nerve can occur, leading to temporary or persistent sensory disturbances. This case report describes a 35-year-old female who sought correction of a small chin. Preoperative evaluation revealed a retrognathic profile, and the patient underwent genioplasty with high-density porous polyethylene implantation. Postoperatively, she experienced mild paresthesia, which improved over time. Neurosensory assessments, including mechanical and light touch tests, showed no abnormalities in A-beta and C fibers but decreased sensitivity in A-delta fibers. This case report emphasizes the importance of evaluating larger nerve fibers during postoperative assessments and the need for standardized testing methodologies to comprehensively assess nerve damage after genioplasty. Further research should explore strategies to standardize neurosensory assessment and optimize therapeutic interventions for nerve damage after genioplasty.

## Introduction

Genioplasty is a common plastic surgery procedure, with over 12,000 interventions performed in the United States in 2015. To achieve satisfactory results, a thorough preoperative evaluation is essential, including managing patient expectations, advanced surgical technique, and selection of appropriate materials [1].

Alloplastic biomaterials like silicone, expanded polytetrafluoroethylene (e-PTFE), Mersilene mesh, and high-density porous polyethylene are commonly used in mandibular plastic surgery, particularly in procedures involving the chin and gonial angle. These materials offer several advantages, including ease of use, elimination of donor site morbidity, and reduced surgical time. They also come in a wide variety of shapes and sizes for precise reconstruction [2].

Silicone and e-PTFE are more malleable, while high-density porous polyethylene offers better graft adaptation and requires less surgical time. However, silicone, despite its biocompatibility and stability, can lead to complications such as encapsulation, migration, late infection, erosion, and bone resorption [3].

Regarding complications, genioplasty can lead to dysfunction in the mentalis muscles and the mental nerve, which is a branch of the inferior alveolar nerve and provides sensation to the mentonian region, mandibular body, lower lip, and gums of the lower front teeth. Lesions in the mentalis muscle can cause chin ptosis and exposure of the lower incision, common in osseous genioplasty. Additionally, the intraoral approach for porous polyethylene prosthesis implantation may result in mental nerve dysfunction, manifesting as neurapraxia. This is the most frequent adverse effect after surgery, but it typically resolves spontaneously within a few weeks [4].

The neurapraxia can occur due to surgical manipulation close to the nerve or compression by implants, leading to symptoms such as tingling, numbness, or burning sensation in the chin and lower lip. Neuropraxia is usually a temporary damage to the myelin sheath while leaving the nerve itself intact, making it a reversible condition but in more severe cases, it may persist and require additional medical intervention. Prevention of these injuries requires detailed anatomical knowledge and care during the surgical procedure. Careful postoperative follow-up is essential to detect and treat any complications that may arise [4-7].

## Case presentation

A 35-year-old female patient sought the private service of Oral and Maxillofacial Surgery and Traumatology in São Paulo, reporting a small chin that bothered her, especially in profile. She had a Class II profile, which was corrected orthodontically. In the anamnesis, she did not report painful complaints related to the face and denied temporomandibular joint (TMJ) pain, clicks, or crepitus. 

She did not present morphological changes in the extraoral physical examination. Intraorally, she had a good occlusion, without morphological alterations, with intact mucosa. Facial analysis showed a retrognathic profile. Preoperative exams were requested, and a prediction tracing was performed, according to facial analysis based on the true vertical line.

In the chin, infiltrative anesthesia was performed intra and extraorally with 2% mepivacaine with 1:100,000 epinephrine, vestibular incision from canine to canine, mucoperiosteal detachment extending to the bone base, implantation of high-density porous polyethylene (Omnipore™, Two-Piece model), medium size with dimensions of 64 mm x 32 mm x 7 mm, and two titanium screws. The polyethylene prosthesis was inserted into the planned site, where it adapted adequately to the chin anatomy, fixed with two screws from the 2.0 system, each 11 millimeters in length.

Vicryl 4-0 sutures were used, and the surgical wounds were closed in two planes, continuously. The procedure took place in an outpatient setting, and shortly after, the patient was discharged. She was prescribed amoxicillin with potassium clavulanate (875/125mg), one tablet every 12 hours for 10 days; metronidazole (400mg), one tablet every eight hours for seven days; profenide (100mg), one tablet every 12 hours for five days; dipyrone (500mg), one tablet every six hours; and Tylex (30mg), one tablet every 12 hours for five days. 

At 15 days postoperatively, satisfactory evolution was observed, with surgical wound without secretions, good healing, progressing to mild paresthesia with tingling characteristic. She denied motor alterations or loss of temperature sensitivity.

During the physical examination, neurosensory assessment was conducted at specific points: the mentonian region (between the canine and central incisor), the lower lip (between the canine and central incisor), and the mandibular body (posterior, middle, and anterior thirds). The patient rated her sensitivity responses on a visual analog scale (VAS), ranging from 0 (no sensitivity) to 10 (normal sensitivity). This instrument is a standard reference for pain assessment, and the following neurosensory tests are validated in previous papers about this subject [4,5,8]. 

A-beta fiber assessment was performed using calibrated brushes, and the light touch used cotton wool. These results are summarized in Table 1.

**Table 1:** Results of the A-beta fiber assessment VAS: visual analog scale

Points	Mechanical evaluation with brush (VAS)	Light touch test (VAS)
Mentonian	8	7
Lower lip	7	6
Mandibular body		
Posterior	10	9
Middle	9	8
Anterior	8	8

Temperature sensation was evaluated using a cold tuning fork, which showed no abnormalities. A two-point discrimination test, assessing A-delta fibers, was conducted before and after surgery, using a 5mm fixed two-point discriminator. The patient correctly identified most points, except for a lapse during the lower lip test, attributed to lack of attention. 

The pin prick test, assessing C fibers and A-delta fibers, involved applying a sharp probe in a quick and pricking manner at the specified points. The patient exhibited normal sensory responses.

**Figure 1:**
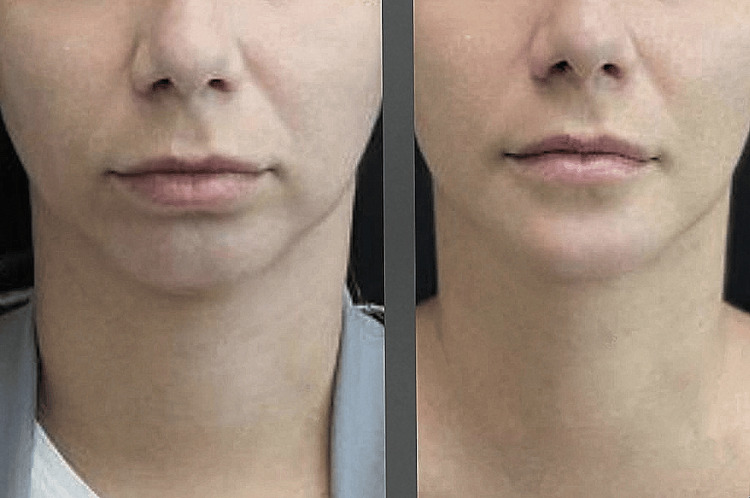
Comparison in frontal view The image on the left shows the patient preoperatively, and the image on the right shows 30 days after surgery.

**Figure 2:**
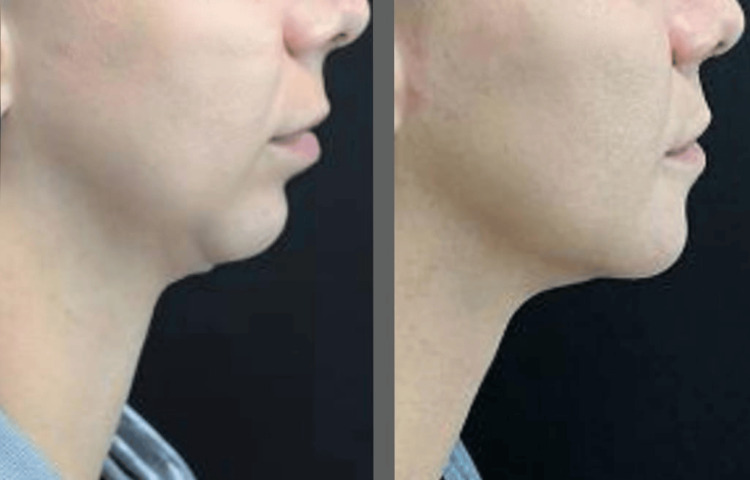
Comparison in profile view The image on the left shows the patient preoperatively, and the image on the right shows 30 days after surgery.

At the 30-day follow-up, the patient showed excellent recovery and was satisfied with the facial profile, achieving the expected outcomes. During the physical examination, no discomfort was reported during the brushing and light touch tests, and the results of the other tests from the initial assessment remained unchanged. The comparison of pre- and post-operative frontal views is available in Figure 1, and the profile view is in Figure 2.

## Discussion

Genioplasty, a surgical procedure to alter the chin’s shape, is widely employed to correct facial deformities and enhance aesthetics. One of the most common complications associated with this procedure is neurapraxia. Neurosensory tests have revealed that approximately 10% of patients who underwent isolated genioplasties experienced altered sensation in the lower lip. Fortunately, this condition tends to resolve in most cases, which aligns with our own case presentation and preventing these injuries requires careful execution during the surgical procedure [8,9]. 

Despite its frequent occurrence, a review indicated that sensory function assessment after genioplasty lacks uniformity, making data comparison challenging. There is a clear need for standardized testing methodologies because delayed treatment may result from this lack of consistency [10].

According to the literature, certain medications, such as anti-inflammatories and vitamin B complex, may be prescribed to manage symptoms and support nerve healing. Complementary therapies, including acupuncture, massage therapy, and biofeedback, are also sometimes used to help manage symptoms and support nerve recovery [11]. 

However, there is a scarcity of randomized, double-blind clinical studies in the current scientific literature to validate the utilization of these treatments. Consequently, despite being a prevalent complication of this surgical procedure, the strategies for treating and assessing this condition are not clearly established [5,12,13]. 

Regarding our results, A-beta and C fibers were spared from damage, while A-delta fibers suffered the most. A study using radiofrequency techniques in trigeminal neuralgia reported that the technique spared A-beta fibers. It concluded that thick nerve fibers, which include these fine fibers within the neural tube, can protect the finest fibers. Additionally, thick fibers are most affected by compression in an animal model of percutaneous trigeminal balloon compression. Furthermore, spontaneous recovery demonstrated in this case report can indicate a favorable prognosis for patients [5,13-16].

## Conclusions

In conclusion, our study supports prior research indicating that larger nerve fibers are particularly susceptible to damage. This highlights the importance of prioritizing the evaluation of larger fibers when assessing nerve damage. Also, this knowledge can lead to more effective therapeutic interventions. Future research should continue to explore strategies to standardize neurosensory assessment.

## References

[1] Choe KS, Stucki-McCormick SU (2000). Chin augmentation. Facial Plast Surg.

[2] Lin J, Chen X (2012). Modified technique of chin augmentation with MEDPOR for Asian patients. Aesthet Surg J.

[3] Patel K, Brandstetter K (2016). Solid implants in facial plastic surgery: potential complications and how to prevent them. Facial Plast Surg.

[4] Degala S, Shetty SK, Bhanumathi M (2015). Evaluation of neurosensory disturbance following orthognathic surgery: a prospective study. J Maxillofac Oral Surg.

[5] de Oliveira RF, Goldman RS, Mendes FM, de Freitas PM (2017). Influence of electroacupuncture and laser-acupuncture on treating paresthesia in patients submitted to combined orthognathic surgery and genioplasty. Med Acupunct.

[6] Kim YK, Kim SG, Kim JH (2011). Altered sensation after orthognathic surgery. J Oral Maxillofac Surg.

[7] Wang Y, Zhang Y, Zhang Z, Li X, Pan J, Li J (2018). Reconstruction of mandibular contour using individualized high-density porous polyethylene (Medpor(®)) implants under the guidance of virtual surgical planning and 3D-printed surgical templates. Aesthetic Plast Surg.

[8] Delgado DA, Lambert BS, Boutris N, McCulloch PC, Robbins AB, Moreno MR, Harris JD (2018). Validation of digital visual analog scale pain scoring with a traditional paper-based visual analog scale in adults. J Am Acad Orthop Surg Glob Res Rev.

[9] Lindquist CC, Obeid G (1988). Complications of genioplasty done alone or in combination with sagittal split-ramus osteotomy. Oral Surg Oral Med Oral Pathol.

[10] Poort LJ, van Neck JW, van der Wal KG (2009). Sensory testing of inferior alveolar nerve injuries: a review of methods used in prospective studies. J Oral Maxillofac Surg.

[11] Manni L, Rocco ML, Barbaro Paparo S, Guaragna M (2011). Electroacupucture and nerve growth factor: potential clinical applications. Arch Ital Biol.

[12] Jafarisavari Z, Ai J, Abbas Mirzaei S, Soleimannejad M, Asadpour S (2024). Development of new nanofibrous nerve conduits by PCL-chitosan-hyaluronic acid containing piracetam-vitamin B12 for sciatic nerve: a rat model. Int J Pharm.

[13] Brown JA, Hoeflinger B, Long PB (1996). Axon and ganglion cell injury in rabbits after percutaneous trigeminal balloon compression. Neurosurgery.

[14] Peters G, Nurmikko TJ (2002). Peripheral and gasserian ganglion-level procedures for the treatment of trigeminal neuralgia. Clin J Pain.

[15] Gregg JM, Banerjee T, Ghia JN, Campbell R (1978). Radiofrequency thermoneurolysis of peripheral nerves for control of trigeminal neuralgia. Pain.

[16] Park JW, Choung PH, Kho HS, Kim YK, Chung JW (2011). A comparison of neurosensory alteration and recovery pattern among different types of orthognathic surgeries using the current perception threshold. Oral Surg Oral Med Oral Pathol Oral Radiol Endod.

